# The lncRNA-AK046375 Upregulates Metallothionein-2 by Sequestering miR-491-5p to Relieve the Brain Oxidative Stress Burden after Traumatic Brain Injury

**DOI:** 10.1155/2022/8188404

**Published:** 2022-02-16

**Authors:** Wei Tang, Weina Chai, Donglin Du, Yongzhi Xia, Yifan Wu, Li Jiang, Chongjie Cheng, Zongduo Guo, Xiaochuan Sun, Zhijian Huang, Jianjun Zhong

**Affiliations:** Department of Neurosurgery, The First Affiliated Hospital of Chongqing Medical University, Chongqing, China 400016

## Abstract

We previously discovered that traumatic brain injury (TBI) induces significant perturbations in long noncoding RNA (lncRNA) levels in the mouse cerebral cortex, and lncRNA-AK046375 is one of the most significantly changed lncRNAs after TBI. lncRNA-AK046375 overexpression and knockdown models were successfully constructed both *in vitro* and *in vivo*. In cultured primary cortical neurons and astrocytes, lncRNA-AK046375 sequestered miR-491-5p, thereby enhancing the expression of metallothionein-2 (MT2), which ameliorated oxidative-induced cell injury. In addition, upregulated lncRNA-AK046375 promoted the recovery of motor, learning, and memory functions after TBI in C57BL/6 mice, and the underlying mechanism may be related to ameliorated apoptosis, inhibited oxidative stress, reduced brain edema, and relieved loss of tight junction proteins at the blood-brain barrier in the mouse brain. Therefore, we conclude that lncRNA-AK046375 enhances MT2 expression by sequestering miR-491-5p, ultimately strengthening antioxidant activity, which ameliorates neurological deficits post-TBI.

## 1. Introduction

Traumatic brain injury (TBI) is a significant cause of morbidity and mortality worldwide [1, 2]. According to its pathophysiological process, TBI can be divided into primary injury and secondary injury. The secondary injury, such as oxidative stress, mitochondrial dysfunction, calcium overloading, and neuroinflammation, strongly influences the outcomes of TBI, which to some degree are reversible and can be mitigated by therapeutics [3–5]. Although there have been many studies seeking to develop TBI therapies in recent years, no available therapeutic method has been confirmed to convey significant improvement for TBI patients in clinical practice.

Long noncoding RNAs (lncRNAs) are defined as RNAs with >200 nucleotides in length without protein-coding ability. lncRNAs have been found to regulate gene expression at the epigenetic, transcriptional, and posttranscriptional levels and actively participate in various physiological and pathological processes [6]. Increasing evidence has shown that lncRNAs participate in the modulation of central nervous system (CNS) development and disease [7]. Several studies have demonstrated that TBI induces perturbations of lncRNA levels in the mouse brain, but few studies have probed the possible roles of lncRNAs in the pathological progression of TBI. We previously reported significant upregulation of lncRNA-AK046375 levels (~4-fold compared to preinjury levels) in the mouse cortex around the injury site after TBI [8], and the present study is aimed at further interrogating whether and how lncRNA-AK046375 is involved in post-TBI pathology. lncRNA-AK046375, with a clone number of “B230377K03” in the GenBank database, has 2,662 nucleotide bps and has not been described by its biological functions. In our lncRNA-AK046375 overexpressing cell model, next-generation sequencing results revealed that lncRNA-AK046375 might be involved in cellular antioxidation activity. Excessive production of oxidants and free radicals is a common feature in the injured cortex after TBI, and relieving oxidative stress burden is expected to be neuroprotective and to ameliorate neurological deficits after TBI.

In this study, lncRNA-AK046375 was found to function as an antioxidative factor, conveying neuroprotection through inhibiting apoptosis, maintaining the integrity of the blood brain barrier (BBB), and relieving brain edema in the mouse brain after TBI. Moreover, the mechanism underlying the antioxidative effects of lncRNA-AK046375 is likely related to its sequestering miR-491-5p and enhancing MT2 expression.

## 2. Materials and Methods

### 2.1. Ethics Statement

All procedures strictly followed the institutional guidelines of Chongqing Medical University and complied with the Guide for the Care and Use of Laboratory Animals issued by the Ministry of Science and Technology of the People's Republic of China. Animal experiments complied with the ARRIVE (Animal Research: Reporting *In Vivo* Experiments) ethical guidelines and were confirmed by the Institutional Animal Care and Use Committee (IACUC) of Chongqing Medical University (approving number: 20141011).

### 2.2. Recombinant Overexpression and Knockdown lncRNA-AK046375 Adenovirus

The lncRNA-AK046375 overexpression adenovirus (1.0 × 10^11^ pfu/ml), overexpression control adenovirus (5.5 × 10^10^ pfu/ml), lncRNA-AK046375 knockdown adenovirus (1.0 × 10^11^ pfu/ml), and knockdown control adenovirus (1.0 × 10^11^ pfu/ml) were produced by Sangon Biotech Co., Ltd. (Shanghai, China). The overexpression of adenovirus constructs is pADV-mCMV-MCS, and the knockdown of adenovirus constructs is pAVsiRNA1.1.

### 2.3. Animals

Adult male C57BL/6 mice (*n* = 270) were purchased from the animal experimental center of Chongqing Medical University (Chongqing, China). Mice were randomly divided into 6 groups: (1) sham (received only craniotomy, but no TBI or any treatment, *n* = 45), (2) TBI (received only TBI, *n* = 45), (3) AK046375 overexpression (received TBI and AK046375-overexpression adenovirus, *n* = 45), (4) vector overexpression (received TBI and AK046375 overexpression control adenovirus, *n* = 45), (5) AK046375 knockdown (received TBI and AK046375 knockdown adenovirus, *n* = 45), and (6) vector knockdown (received TBI and AK046375 knockdown control adenovirus, *n* = 45). All mice were housed in the experimental animal center of Chongqing Medical University (Chongqing, China) in a 12/12 dark and light environment. Methods of anesthesia induction were followed as in our previously published study [9]. Newborn mice (*n* = 120) were used to extract and culture primary cortical neurons and astrocytes.

### 2.4. Intracerebroventricular Injection

The adenovirus was cranially injected into the lateral ventricles in a stereotaxic device under general anesthesia based on protocols described in our previously published study [9]. Briefly, once achieving an appropriate level of anesthesia (the mice in this study were anesthetized induction with 3% isoflurane in a 67% N_2_O/30% O_2_, and maintained by 5% isoflurane), 3 *μ*l diluted adenovirus were injected into the right cerebral lateral ventricle at the following coordinates (mm from the bregma) under stereotaxic apparatus guidance: AP+1.5, ML+1, and DV-2.

### 2.5. Controlled Cortical Injury (CCI)

A CCI model was exploited to mimic TBI in mice under appropriate general anesthesia on 7 days after adenovirus injection using our previously reported parameters: (1) velocity: 5.0 m/s, [2] depth: 2.0 mm, and [3] dwelling time: 100 ms. The sham group received only a craniotomy, but no CCI impact [9].

### 2.6. Neurobehavioral Tests

We evaluated neurobehaviors in mice according to our previously published protocols [9, 10]. Briefly, neurological severity scores (NSS), wire-gripping scores, and rotarod test were used to evaluate neurological function in mice on the preoperative, 1, 3, 7, and 14 days after CCI. Cued learning ability was assessed using the Morris water maze test on 15 to 19 days after CCI. The spatial memory was evaluated by Morris water maze test on 20 days after CCI when the hiding platform was removed. The platform was located in quadrant 4.

### 2.7. Cell Culture

#### 2.7.1. HT22 Cell Culture

The immortalized mouse hippocampal cell line (HT22) was provided by JENNIO Biological Technology (Guangzhou, China) and cultured in DMEM (Gibco, Carlsbad, USA) with 10% fetal bovine serum (FBS) (Wisent Biotechnology Co. Ltd., Nanjing, China) and 1% penicillin and streptomycin (Gibco, Carlsbad, USA). And the culture media was regularly replaced every 3 days.

#### 2.7.2. Primary Cortical Neuron Culture

Primary cortical neurons were harvested from newborn mice (C57BL/6, postnatal 24 h) as described in previous studies [11]. Briefly, the newborn mice were disinfected with 75% ethanol and euthanized by decapitation, and the meninges were removed out and the cerebral cortex was made into single suspended cells with digesting buffer (papain (2 mg/ml)) (Worthington, America)/DNase I (Solarbio life science, Beijing, China) (10 mg/ml) = 1/100). The cells were seeded into the plates coated with polylysine (Solarbio Life Science, Beijing, China). Culture media (Neurobasal-A (Gibco, Carlsbad, USA) containing 2% B27 (Invitrogen, America) and 2 Mm glutamine (Gibco, Carlsbad, USA)) were regularly replaced every 3 days, and adenovirus were used to infect neurons at a MOI = 50 for 8 h.

#### 2.7.3. Primary Cortical Astrocyte Culture

Primary cortical astrocytes were harvested using previously described protocols [12, 13]. Briefly, the newborn mice were disinfected with 75% ethanol and euthanized by decapitation, and the meninges were removed out, and the cerebral cortex was made into single suspended cells with digesting buffer (0.025%Tyrisin/DNase I (10 mg/ml) = 1/100). Adenovirus was used to infect the astrocytes under an MOI = 20 for 6 h. Media was replaced with complete medium (DMEM/F12 : FBS = 10 : 1) after miR-491-5p mimics, miR-491-5p inhibitor, mimics-NC, and inhibitor-NC (Gene Pharma, Shanghai, China) were diluted in DMEM/F12 medium to transfect astrocytes for 7 h. To investigate how AK046375 regulates MT2 expression, miR-491-5p mimic or inhibitor was administered for 48 h starting on the 3^rd^ day after virus infection.

### 2.8. Cell Counting Kit 8 (CCK8) Assay

The CCK8 assay (Dojindo Laboratories, Japan) was performed per the instructions. In brief, primary cortical neurons and astrocytes were washed with PBS after received H_2_O_2_ treatments (supplementary fig. 3); 100 *μ*l of serum-free medium containing 10% CCK8 working buffer was added to each well and maintained (neuron for 4 h, astrocyte for 2 h) at 37°C. A microplate reader (Thermo Fisher, America) was used to detect the absorbance at the 550 nm. The cell vitality was calculated by the ratio of (sample group − blank group)/(nontreated group − blank group).

### 2.9. LDH Assay

Lactate dehydrogenase (LDH) released into the medium from cells was measured using the LDH release assay kit (Beyotime biotechnology, Shanghai, China) as described in instructions. The LDH releasing rate was calculated by the ratio of (sample group − blank group)/(nontreated group − blank group).

### 2.10. Quantitative Real-Time Polymerase Chain Reaction (qRT-PCR) Assay

TRIzol reagent (Takara, Japan) was applied to extract total cellular or tissue RNA. qRT-PCR was applied on an Applied Biosystems® 7500 Fast system. mRNA primers (AK046375: forward: GCTCAAGGGATATGGCCAGC, reverse: CGCTCAGCTCCTTTGCTCTC; MT2: forward: CAGATATACCAGCCCAGTGAAC, reverse: GAG- TGTACTTGGTAGAGGTGAC; miR-491-5p: forward AGTGGGGAACCCTTCCATGAG) were synthesized by Sangon Biotech (Shanghai, China) and normalized to actin and U6, respectively. The quantification of genes was carried out per the formula 2^-(*Δ*Ct-*Δ*Ct)^.

### 2.11. Western Blotting Assay

Tissue and cell lysates were separated, transferred to 0.45 *μ*m PVDF membranes (Millipore, America), blocked and incubated in primary antibodies (metallothionein 2, MT2, 1 : 200, Immunoclone, America, ICA868Hu01). BCL2(1 : 1000, 15071), cleaved-caspase 3 (1 : 1000, 9661S), SOD2(1 : 1000, 13194), CAT (1 : 1000, 12980) (CST, America). cytochrome C (1 : 500, Abcam, Britain, ab133504). Bax (1 : 1000, 60267-1-Ig), VDAC1(1 : 1000, 66345-1-Ig) (Proteintech, Wuhan, China), malondialdehyde (MDA) (1 : 1000, Novus, America, NBP2-59366). claudin 5 (1 : 1000, #AF5216), ZO1 (1 : 1000, #AF5145), occludin (1 : 1000, #DF7504) (Affinity, Jiangsu, China), and *β*-actin (1 : 800, Boster, Wuhan, China, BA2305), and followed by incubation in HRP-conjugated secondary antibodies (Proteintech, Wuhan, China, SA00001-1 and SA00001-2). The membranes were visualized by the ECL system (Thermo, America, 32132), and the quantification of the band intensity were calculated by the ImageJ software [14, 15].

### 2.12. GSH and GSSG Assays

The total glutathione/oxidized glutathione assay kit (Nanjing JianCheng, Nanjing, China) was used to detect expression GSSG (oxidized glutathione, GSSG), GSH (reduced glutathione), and the GSH/GSSG ratio per the manufacturer's protocol.

### 2.13. MitoSOX™ Assay

The MitoSOX™ red mitochondrial superoxide indicator (Invitrogen, America) was used to determine mitochondrial superoxide expression in neurons and astrocytes as previously described [16]. Briefly, the live cells were incubated with 5 *μ*M of MitoSOX™ Red at 37°C for 10 min after received the treatments and then washed 3 times in Hank's solution; the stained cells were imaged by confocal microscopy with analysis using Image-Pro Plus.

### 2.14. Immunofluorescence Assay

Immunofluorescence staining was performed as described in previous protocols [15]. Brain sections, primary cortical neurons, and astrocytes were fixed in 4% paraformaldehyde, blocked in 5% goat serum, and then incubated in primary antibody *β*3 tubulin (1 : 50, 4466), GFAP (1 : 400, 3670), NeuN (1 : 400, #243075), (CST, America), and MT2 (1 : 25) at 4°C overnight, followed by incubation with secondary antibody (dylight 650 goat anti-mouse IgG (A23610), dylight 594 goat anti-rabbit IgG (A23420)) (Abbkine, America) at 37°C for 60 min. Images were taken by confocal microscopy.

### 2.15. TUNEL Assay

Apoptotic cells were detected as in previously published protocols [17]. Briefly, the one step TUNEL apoptosis assay kit (Beyotime, Shanghai, China) was performed per the manufacturer's instructions, and images were taken by confocal microscopy.

### 2.16. Brain Water Content Assay

Brain water content was assessed on 7 days after CCI as previously described and calculated as [(wet weight–dry weight)/wet weight] × 100%^2^.

### 2.17. Evans Blue Fluorescence and Extravasation of Evans Blue In *Vivo*

Evans blue fluorescence and the content of Evans blue in brain tissue on 7 days after TBI were performed as in previous protocols [2, 18]. Briefly, mice were injected with 2% Evans blue (5 ml/kg) via the tail vein 1 h before brain removal, and the brain was snap frozen right after removal and sectioned and visualized by confocal microscopy. Besides, we also detected the content of Evans blue in brain tissue which exuated from the injury vascellum. The brain supernatants were made with 0.9% saline after perfusion with 0.9% saline; the contents of Evans blue in mice were detected by spectrophotometer.

### 2.18. Dual-Luciferase Reporter Assay

The dual-luciferase reporter assay was performed as previously published to determine whether miRNAs directly bind to their target gene. Briefly, to verify whether miR-491-5p and/or miR-505-3p could bind to the 3′-UTRs of MT2-mRNA and AK046375, the predicted 3′-UTRs wild-type or mutant binding sequence of MT2 or AK046375 were designed and predicted using the databases “miRanda,” “PITA,” and “RNAhybrid.” Binding sequences (wild-type or mutant binding sequences) were cloned into the pmirGLO vector. Firstly, the pGL3-MT2-WT vector, pGL3-MT2-MUT vector, miR-491-5p/miR-505-3p mimics, and its negative controls were randomly cotransfected into 293T cells to verify whether miR-491-5p and/or miR-505-3p could bind to the 3′-UTRs of MT2-mRNA. Secondly, the pGL3-AK046375-WT vector, pGL3-AK046375-MUT vector, miR-491-5p mimics, and its negative control were randomly cotransfected into 293T cells to verify whether AK046375 could sequester miR-491-5p. And then, a chemiluminescent analyzer (Biosino, Beijing, China) was used to detected luciferase activity.

### 2.19. RNA Immunoprecipitation (RIP) Assay

The RIP assay was performed per the manufacturer's instructions. In brief, HT22 cells were lysed in 500 *μ*l RIP lysis buffer (Thermo Fisher, America). Normal mouse IgG was regarded as a negative control and was separately added into the RIP buffer. Dynabeads® protein G (Thermo, America) were then added into the buffer, collected, and divided into two parts, one for detecting the content of AGO2 (CST, America) by western blotting and the remaining for detecting the expression levels of AK046375 and miR-491-5p by qRT-PCR.

### 2.20. Statistical Analysis

All experimental data were analyzed by SPSS 19.0 software and are expressed as the mean ± standard deviation (SD). NSS scoring and water maze test (latency and time spent in the correct quadrant (%)) were analyzed by two-way ANOVA followed by Tukey's post hoc test [9]. The remaining data were analyzed using *t*-test or one-way ANOVA followed by Tukey's post hoc test. All statistical plots were created using GraphPad Prism V5.0, and *P* < 0.05 was considered statistically significant.

## 3. Results

### 3.1. AK046375 Overexpression Induces Significantly Increased MT2 Expression

**Figure 1:**
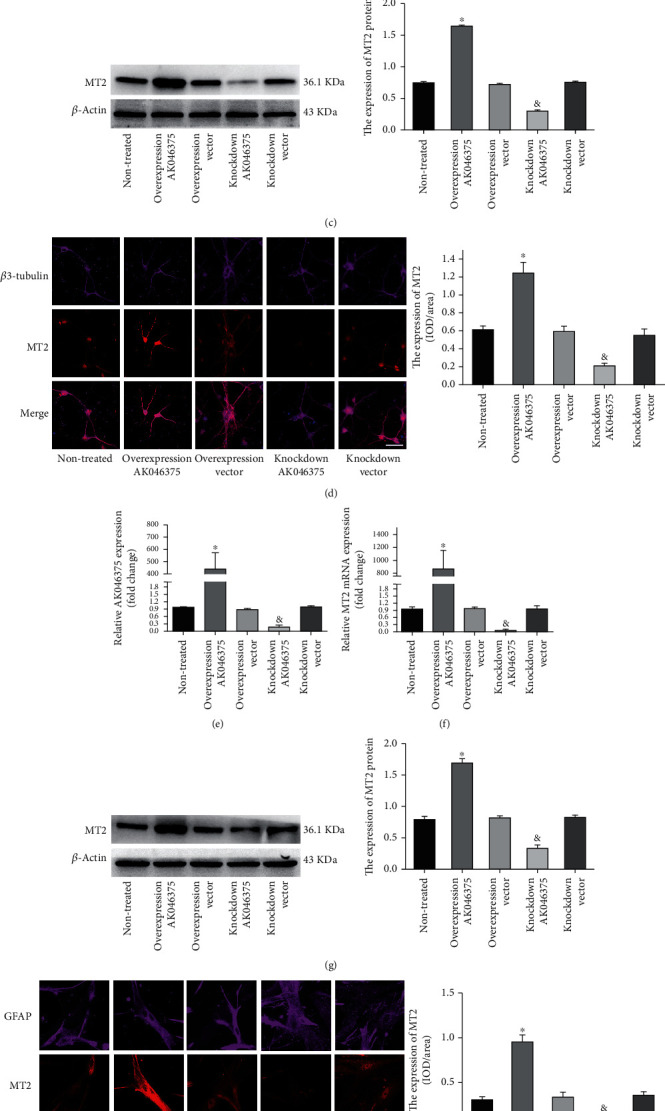
AK046375 significantly upregulates the expression of MT2 in primary cortical neurons and astrocytes. (1) Quantitative PCR analysis of AK046375 levels in mouse primary cortical neurons (a) and astrocytes (e) after transfection with either overexpression or knockdown AK046375 adenovirus or their responding control vector viruses. (2) Quantitative PCR analysis of MT2-mRNA levels and western blotting results of MT2 in mouse primary cortical neurons (b, c) and astrocytes (f, g) after transfection with either overexpression or knockdown AK046375 adenovirus or their corresponding vector viruses. (3) Immunofluorescence images of MT2 in primary neurons (d) and astrocytes (h) with different AK046375 levels and their quantification. *β*3-Tubulin is a neuronal marker, and glial fibrillary acidic protein (GFAP) is an astrocytic marker (*n* = 6/group, mean ± SD, ^∗^*P* < 0.05 vs. the overexpression vector group, ^&^*P* < 0.05 vs. the knockdown vector group by one-way ANOVA; scale bar = 50 *μ*m, 400x).

We used 5′- and 3′-RACE analyses to identify a 2581 bp full-length transcript of AK046375 (supplementary figure 1) and performed RNA-seq followed by bioinformatics analysis to determine whether overexpression of lncRNA-AK046375 induced alterations in mRNAs. Our next-generation sequencing results revealed that 1342 mRNAs were significantly altered in response to increased AK046375 overexpression (supplementary fig. 2A). These raw data were all submitted to the GEO database (ID number: GSE103353). Among the most significantly changed genes, we noticed that MT2 mRNA level was significantly elevated (~6-fold) by AK046375 overexpression (supplementary table 1). In addition to upregulation of MT2 mRNA, MT2 protein levels were also significantly increased in HT22 cells by AK046375 overexpression (supplementary fig. 2B). To further prove that AK046375 could upregulate the expression of MT2, we also tested whether AK046375 could upregulate the MT2 expression in primary cortical neurons and astrocytes. Firstly, we succeeded to transfect the AK046375 overexpression and knockdown adenovirus into the primary cortical neurons and astrocytes (Figures 1(a) and 1(e)). Secondly, it was interesting to found that AK046375 overexpression could also significantly increase the expression of MT2 compared to the nontreated group, by qRT-PCR, western blotting, and immunofluorescence in primary cortical neurons (*P* < 0.05, Figures 1(b)–1(d)) and astrocytes (*P* < 0.05, Figures 1(f)–1(h), and downregulation of AK046375 could significantly decrease the MT2 expression compared to the nontreated group in primary cortical neurons (*P* < 0.05, Figures 1(b)–1(d)) and astrocytes (*P* < 0.05, Figures 1(f)–1(h)). Therefore, these results proved that AK046375 overexpression resulted in upregulation of MT2 expression, suggesting that MT2 might be involved in the biological functions of AK046375. MT2 is a special protein with low molecular weight and high cysteine that binds a large number of heavy metal ions, which grant it strong antioxidant activity [19, 20].

Given these results, we hypothesized that upregulated AK046375 in the cerebral cortex after TBI might be involved in cellular antioxidation activity. Therefore, we investigated the antioxidation effects of AK046375 and the underlying mechanism.

### 3.2. Potential Mechanisms by which AK046375 Increases MT2 Expression

#### 3.2.1. miR-491-5p Decreases MT2 Expression and Exaggerates H_2_O_2_-Induced Oxidative Stress in Astrocytes

**Figure 2:**
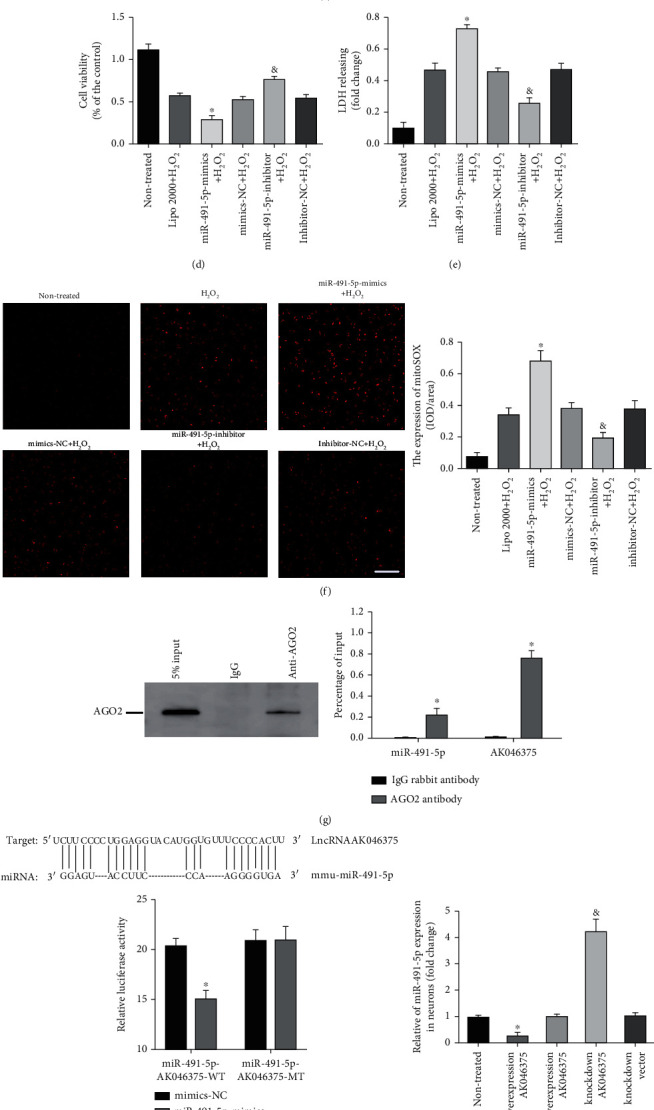
AK046375 upregulates MT2 expression by acting as a “sponge” for miR-491-5p. (a) Predicted binding sites of miR-491-5p and miR-505-3p with MT2-mRNA-3′-UTR and dual-luciferase reporter gene assay results verifying their binding activity (*n* = 5/group, mean ± SD, ^∗^*P* < 0.05 vs. the mimics-NC group by *t*-test). (b) Expression of MT2 mRNA and MT2 protein levels in response to miR-491-5p-mimics or miR-491-5p-inhibitors in primary astrocytes (*n* = 6/group, mean ± SD, ^∗^*P* < 0.05 vs. the mimics-NC group, ^&^*P* < 0.05 vs. the inhibitors-NC group by one-way ANOVA). (d–f) Cell viability, membrane damage, and oxidative burden in astrocytes of each group (*n* = 6/group, mean ± SD, ^∗^*P* < 0.05 vs. the mimics-NC+H_2_O_2_ group, ^&^*P* < 0.05 vs. the inhibitors-NC+H_2_O_2_ group by one-way ANOVA, scale bar = 50 *μ*m, 200x). (g) Ago2 protein level detected by western blot analysis. Quantitative PCR analysis of AK046375 and miR-491-5p levels compared to the input (*n* = 3/group, mean ± SD, ^∗^*P* < 0.05 vs. the IgG group by *t*-test). (h) Potential binding sites of miR-491-5p with AK046375 (up), and luciferase activity of the wild-type and mutant AK046375 groups with miR-491-5p in 293T cells (down) (*n* = 5/group, mean ± SD, ^∗^*P* < 0.05 vs. the mimics-NC group by *t*-test). (i, j) Quantitative PCR analysis of miR-491-5p levels in primary neurons and astrocytes after transfection with either overexpression or knockdown AK046375 adenovirus or their corresponding vector viruses (*n* = 6/group, mean ± SD, ^∗^*P* < 0.05 vs. the overexpression vector group, ^&^*P* < 0.05 vs. the AK046375 overexpression group by one-way ANOVA). (k, l) Quantitative PCR analysis of MT2 mRNA levels and western blotting results of MT2 in astrocytes from each group after transfection with AK046375 overexpression or knockdown adenovirus, miRN-491-5p-mimics, and miR-491-5p-inhibitor and their quantification (*n* = 4/group, mean ± SD, ^∗^*P* < 0.05 vs. the AK046375 overexpression group, ^&^*P* < 0.05 vs. the AK046375 knockdown group by one-way ANOVA).

miRNAs are small noncoding RNAs (18-23 bp) that inhibit the translation of their target mRNAs by binding to the 3′-UTR of mRNA. Both AK046375 and MT2 mRNA were predicted to possess common binding sites for miR-491-5p and miR-505-3p. However, in our dual-luciferase reporter assay, only miR-491-5p, not miR-505-3p, decreased MT2 wild-type fluorescence intensity (*P* < 0.05, Figure 2(a)), indicating that miR-491-5p does indeed bind to the 3′-UTR of MT2 mRNA directly. We also found that MT2 mRNA and protein expression levels were higher in the miR-491-5p inhibitor group (*P* < 0.05, Figures 2(b) and 2(c)) and lower in the miR-491-5p mimics group (*P* < 0.05, Figures 2(b) and 2(c)) compared to their respective control groups, indicating that miR-491-5P indeed decreases the expression of MT2.

When H_2_O_2_ treatment was applied in primary astrocytes, cell vitality was reduced in the miR-491-5p mimics+H_2_O_2_group (*P* < 0.05, Figure 2(d)) but was enhanced in the miR-491-5p inhibitor+H_2_O_2_ group (*P* < 0.05, Figure 2(d)) compared to the inhibitor-NC+H_2_O_2_ group and the mimics-NC+H_2_O_2_ group, respectively. The content of LDH and fluorescence intensity of MitoSOX^red^ were higher in the miR-491-5p mimics+H_2_O_2_ group (*P* < 0.05, Figures 2(e) and 2(f)) compared to the mimics-NC+H_2_O_2_ group, but lower in the miR-491-5p inhibitor+H_2_O_2_ group (*P* < 0.05, Figures 2(e) and 2(f)) compared to the inhibitor-NC+H_2_O_2_ group. These results indicate that miR-491-5p exaggerates H_2_O_2_-induced injury in astrocytes by inhibiting the expression of MT2.

#### 3.2.2. AK046375 Enhances MT2 Expression by Sequestering miR-491-5p

To confirm that AK046375 upregulated MT2 expression by competitively sequestering miR-491-5p, we performed a dual-luciferase reporter assay and found that miR-491-5p decreased the fluorescence intensity of the AK046375 wild-type group significantly (*P* < 0.05, Figure 2(h)), indicating that miR-491-5p can bind to the AK046375. miRNAs have been found to perform their functions through forming ribonucleoprotein complexes (miRNPs) in which AGO2 is involved as a core component [21]. An RIP assay was performed in HT22 cells and demonstrated that both AK046375 and miR-491-5p were pulled down by the AGO2 antibody (Figure 2(g)), Furthermore, AK046375 overexpression could also inhibit the expression of miR-491-5p (*P* < 0.05, Figures 2(i) and 2(j)) in primary cortical neurons and astrocytes compared to their respective control groups.

To further investigate the relationship of AK046375 and miR-491-5p in the regulation of MT2 expression, we found that the expression of MT2 was lower in the AK046375 overexpression+miR-491-5p mimics group (*P* < 0.05, Figures 2(k) and 2(l)) compared to the AK046375 overexpression group and was higher in the AK046375 knockdown+miR-491-5p inhibitor group (*P* < 0.05, Figures 2(k) and 2(l)) compared to the AK046375 knockdown group, indicating that miR-491-5p is involved in the modulation of AK046375 on the MT2 expression. Based on AK046375 being simultaneously pulled down with miR-491-5p and miR-491-5p imposing a direct mediating effect on AK046375 for MT2 regulation, we inferred that AK046375 might upregulate MT2 expression by sequestering miR-491-5p.

### 3.3. AK046375 Alleviates H_2_O_2_-Induced Injury in Primary Cortical Neurons and Astrocytes

#### 3.3.1. AK046375 Alleviates H_2_O_2_-Induced Oxidative Stress in Primary Cortical Neurons and Astrocytes

**Figure 3:**
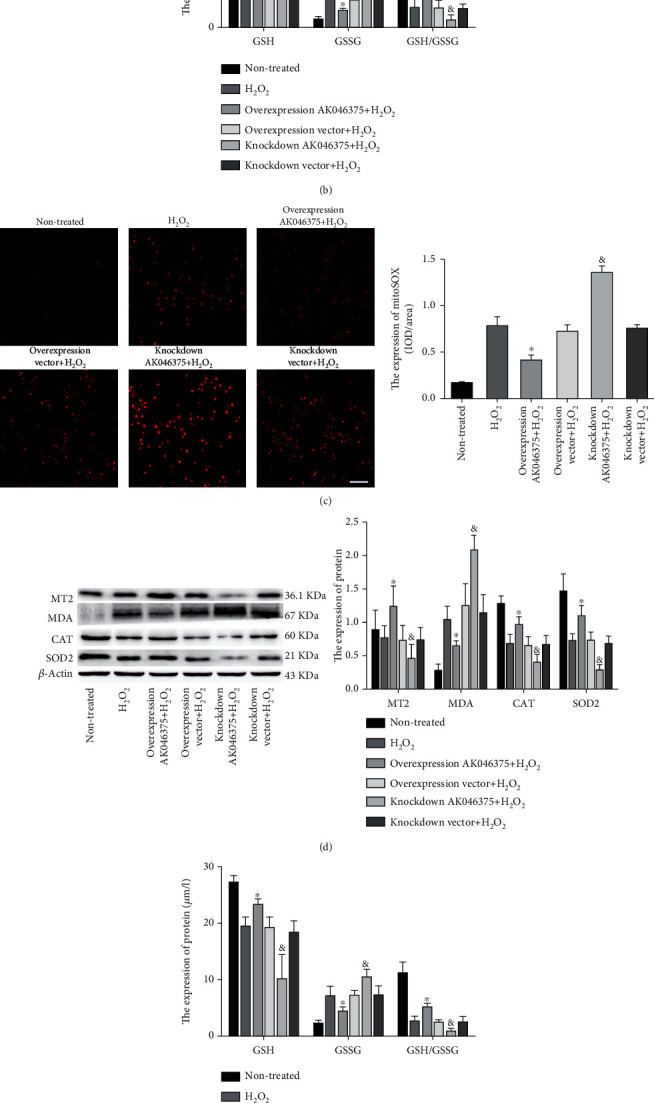
AK046375 alleviates H2O2-induced oxidative stress in primary neurons and astrocytes. (1) Western blotting images of MT2, MDA, CAT, and SOD2 in neurons (a) and astrocytes (d) for each group, and the content of GSH, GSSG, and GSH/GSSG in neurons (b) and astrocytes (e) with quantification. (2) Fluorescence of MitoSOX^red^ in neurons (c) and astrocytes (f) and quantification (scale bar = 50 *μ*m, 400x for neurons; scale bar = 50 *μ*m, 200x for astrocytes) (*n* = 6/group, mean ± SD, ^∗^*P* < 0.05 vs. the overexpression vector+H_2_O_2_ group, ^&^*P* < 0.05 vs. the knockdown vector+H_2_O_2_ group by one-way ANOVA).

We measured oxidative stress in cells in response to H_2_O_2_ treatment. MitoSOX^red^ staining was applied to specifically detect reactive oxygen species in mitochondria. CAT, GSH, and SOD2 play important roles in cellular against the oxidative injury, MDA play a vital role in cellular oxidative injury. To explore whether the AK046375 decreased the oxidative stress level in cellular under H_2_O_2_ treatment, we detected the expression of CAT, SOD2, GSH, GSH/GSSG, MDA, GSSG and the MitoSOX red staining fluorescence intensity in cellular. H_2_O_2_ led the decrease expression of CAT, GSH, and SOD2 and increased the production of MDA compared to the nontreated group (*P* < 0.05, Figures 3(a) and 3(d)). There was no significant difference in the expression of CAT, SOD2, MT2, GSH, GSH/GSSG, MDA, GSSG, or the MitoSOX^red^ staining fluorescence intensity among the H_2_O_2_, overexpression vector, and knockdown vector groups (*P* > 0.05, Figure 3). The expressions of CAT, SOD2, MT2, GSH, and GSH/GSSG were higher in the AK046375 overexpression group (*P* < 0.05, Figures 3(a), 3(b), 3(d), and 3(e)) but lower in the AK046375 knockdown group (*P* < 0.05, Figures 3(a), 3(b), 3(d), and 3(e)) compared to their respective control groups. Expression of MDA, GSSG, and MitoSOX^red^ staining fluorescence intensity were lower in the AK046375 overexpression group (*P* < 0.05, Figure 3) and higher in the AK046375 knockdown group (*P* < 0.05, Figure 3) compared to their respective control groups. These results indicate that AK046375 alleviates H_2_O_2_-induced oxidative stress in primary neurons and astrocytes.

#### 3.3.2. AK046375 Decreases H_2_O_2_-Induced Apoptosis in Primary Cortical Neurons and Astrocytes

**Figure 4:**
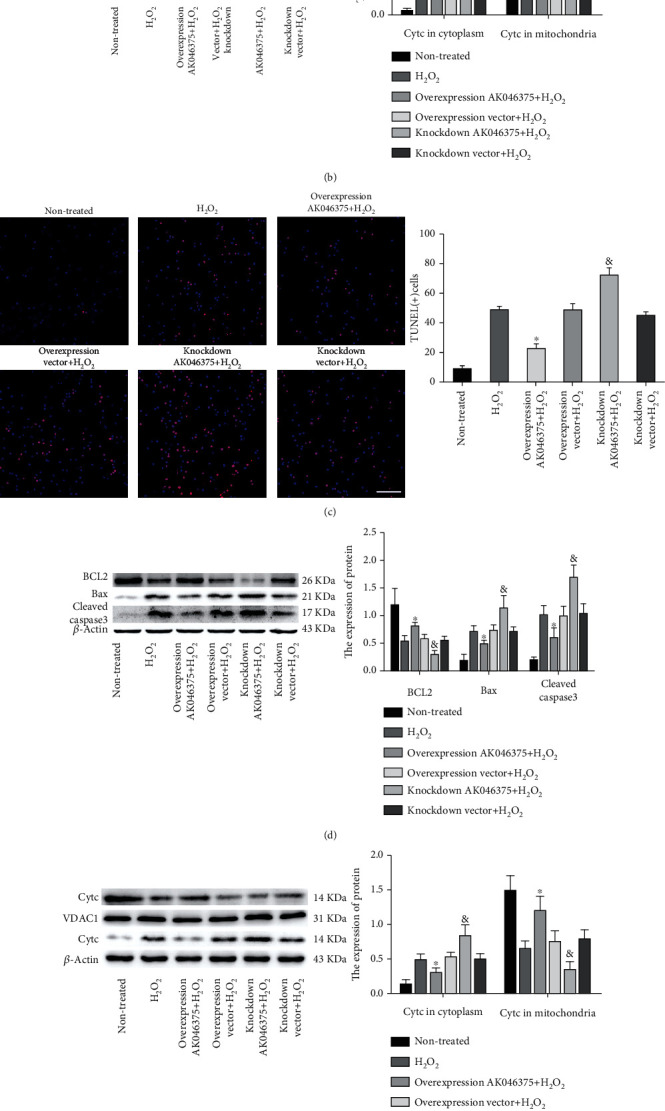
AK046375 decreased the H_2_O_2_-induced apoptosis on primary cortical neurons and astrocytes. (1) BCL2, Bax, and cleaved-caspase-3 in neurons (a) and astrocytes (d) detected by western blotting and quantification. (2) Cytochrome C (Cytc) in cytoplasm and mitochondria in neurons (b) and astrocytes (e) and quantification (VDAC1 was used as a loading control for mitochondrial proteins.). (3) TUNEL-positive cells in neurons (c) and astrocytes (f) and quantification (scale bar = 50 *μ*m, 400x for neurons; scale bar = 50 *μ*m, 200x for astrocytes) (*n* = 6/group, mean ± SD, ^∗^*P* < 0.05 vs. the overexpression vector group, ^&^*P* < 0.05 vs. the knockdown vector group by one-way ANOVA).

The proteins of the BCL-2 family are the most important regulators of mitochondria-related apoptosis. The ratio of pro- and antiapoptotic proteins (BCL2 and Bax) determines whether mitochondria initiate the cell death program by releasing cytochrome C and other proapoptotic factors [22]. There was no significant difference in the number of TUNEL-positive cells or in the expression of BCL2, Bax, and cleaved-caspase-3 between the H_2_O_2_, overexpression vector, and knockdown vector groups (*P* > 0.05, Figure 4). The number of TUNEL-positive cells and expression of cleaved-caspase-3 and Bax were significantly lower in the AK046375 overexpression group and higher in the AK046375 knockdown group (*P* < 0.05, Figure 4) compared to their respective control groups. The expression of BCL2 was significantly higher in the AK046375 overexpression group and lower in the AK046375 knockdown group (*P* < 0.05, Figures 4(a) and 4(d)) compared to their respective control groups. Moreover, mitochondrial membrane damage induced the release of cytochrome C from the mitochondria to the cytoplasm and decreased the release inhibited apoptosis [23]. No significant differences were found in mitochondrial or cytoplasmic cytochrome C levels among the H_2_O_2_, overexpression vector, and knockdown vector groups (*P* > 0.05, Figures 4(b) and 4(e)). Expression of cytochrome C in mitochondria was higher in the AK046375 overexpression group and lower in the AK046375 knockdown group (*P* < 0.05, Figures 4(b) and 4(e)) compared to their respective control groups. In addition, cytoplasmic cytochrome C was decreased in the AK046375 overexpression group (*P* < 0.05, Figures 4(b) and 4(e)) and increased in the AK046375 knockdown group (*P* < 0.05, Figures 4(b) and 4(e)) compared to their respective control groups. Besides, we also found that AK046375 could improve the cell survival under the H_2_O_2_ treatment (supplementary fig. 5). These results indicate that AK046375 relieves H_2_O_2_-induced apoptosis in neurons and astrocytes.

### 3.4. AK046375 Exerts Neuroprotective Effects in Mice after TBI

#### 3.4.1. AK046375 Alleviates Oxidative Stress in Mice after TBI

**Figure 5:**
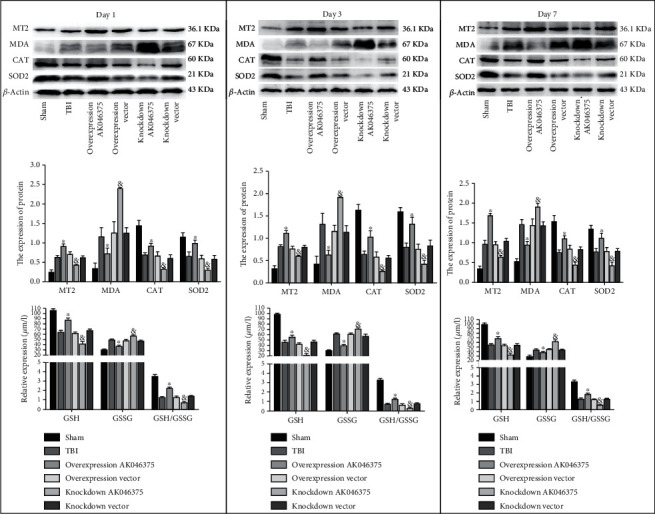
AK046375 alleviates oxidative stress on 1, 3, and 7 days after TBI. Western blotting results of MT2, MDA, CAT, SOD2, and GSSG, GSH, and GSH/GSSG content in each group and quantification after TBI (*n* = 4/group, mean ± SD, ^∗^*P* < 0.05 vs. the overexpression vector group, ^&^*P* < 0.05 vs. the knockdown vector group by one-way ANOVA).

In these *in vivo* experiments, AK046375 overexpression and knockdown adenoviruses were found to efficiently up- and downregulate AK046375 levels in the mouse brain, respectively (supplementary fig. 6A). MT2 mRNA and protein levels were upregulated and downregulated in response to AK046375 overexpression and knockdown groups, compared to their respective control groups (supplementary fig. 6B and C). Expression of GSH, GSSG, GSH/GSSG, MDA, MT2, SOD2, and CAT was not significantly different among the TBI, overexpression vector, and knockdown vector groups (*P* > 0.05, Figure 5). Expression of GSSG and MDA was significantly increased in the AK046375 knockdown group (*P* < 0.05, Figure 5) and decreased in the AK046375 overexpression group (*P* < 0.05, Figure 5) compared to their respective control groups. Expression of GSH, GSH/GSSG, SOD2, MT2, and CAT were significantly decreased in the AK046375 knockdown group (*P* < 0.05, Figure 5) and increased in the AK046375 overexpression group (*P* < 0.05, Figure 5) compared to their respective control groups. These results show that AK046375 alleviates the oxidative stress in the cerebral cortex around the injury focus after TBI.

#### 3.4.2. AK046375 Significantly Decreases Apoptosis and Promotes the Neuron Survival in the Cerebral Cortex of Mice after TBI

**Figure 6:**
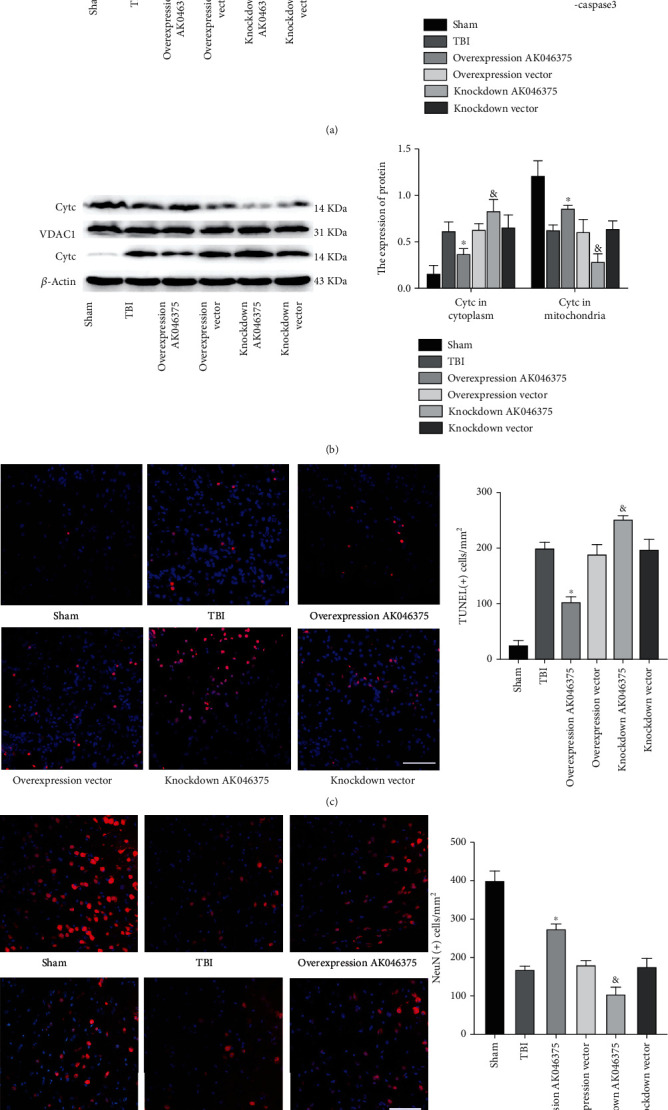
AK046375 inhibits apoptosis on 7 days after TBI. (a) Western blotting results of BCL2, Bax, and cleaved-caspase-3 in each group and quantification. (b) Western blotting results of cytochrome C (Cytc) in cytoplasm (up) and mitochondria (down) and quantification. (c) TUNEL-positive cells in the mouse cortex around the injury site and quantification (scale bar = 50 *μ*m, 400x). (d) NeuN-positive cells in the mouse cortex around the injury site and quantification (scale bar = 50 *μ*m, 400x). NeuN is a neuronal marker. (*n* = 5/group, mean ± SD, ^∗^*P* < 0.05 vs. the overexpression vector group, ^&^*P* < 0.05 vs. the knockdown vector group by one-way ANOVA).

We also detected apoptosis in the cerebral cortex around the injury site 7 days after TBI. The expression of BCL2 was decreased; the Bax, cleaved-caspase-3, and the TUNEL-positive cells were increased in the TBI group compared with the sham group (*P* < 0.05, Figures 6(a) and 6(c)). The expression of BCL2, Bax, cleaved-caspase-3, and the TUNEL-positive cells showed no significant differences among the TBI, overexpression vector, and knockdown vector groups (*P* > 0.05, Figures 6(a) and 6(c)). The expression of BCL2 was significantly increased in the AK046375 overexpression group and decreased in the knockdown group (*P* < 0.05, Figure 6(a)) compared to their respective control groups. The expressions of Bax and cleaved-caspase-3 and TUNEL-positive cells in the overexpression AK046375 group were reduced (*P* < 0.05, Figures 6(a) and 6(c)) compared to the overexpression vector group, and expressions of Bax and cleaved-caspase-3 in the AK046375 knockdown group were increased (*P* < 0.05, Figures 6(a) and 6(c)) compared to the knockdown vector group. TBI caused the release of cytochrome C from mitochondria to the cytoplasm compared to the sham group (*P* < 0.05, Figures 6(a) and 6(c)). The cytochrome C in the mitochondria and cytoplasm showed no significant difference among the TBI, overexpression vector, and knockdown vector groups (*P* > 0.05, Figure 6(b)). AK046375 overexpression significantly decreased cytochrome C release from the mitochondria into the cytoplasm (*P* < 0.05, Figure 6(b)) but AK046375 knockdown significantly increased the release (*P* < 0.05, Figure 6(b)) compared to their respective control groups.

To further confirm the *in vivo* antiapoptosis effect of AK046375, we also quantified the remaining neurons in the cerebral cortex around the injury site on 7 days after TBI. The number of NeuN-positive cells in the TBI group was decreased compared to the sham group (*P* < 0.05, Figure 6(d)). There was no significant difference in the number of NeuN-positive cells between the TBI, overexpression vector, and knockdown vector groups (*P* < 0.05, Figure 6(d)). The number of NeuN-positive cells were higher in the AK046375 overexpression group (*P* < 0.05, Figure 6(d)) and lower in the AK046375 knockdown group (*P* < 0.05, Figure 6(d)) compared to their respective control groups. These results suggest that AK046375 significantly decreases apoptosis and promotes the neuronal survival in the cerebral cortex around the injury site after TBI.

#### 3.4.3. AK046375 Maintains BBB Integrity and Decreases Brain Edema after TBI

**Figure 7:**
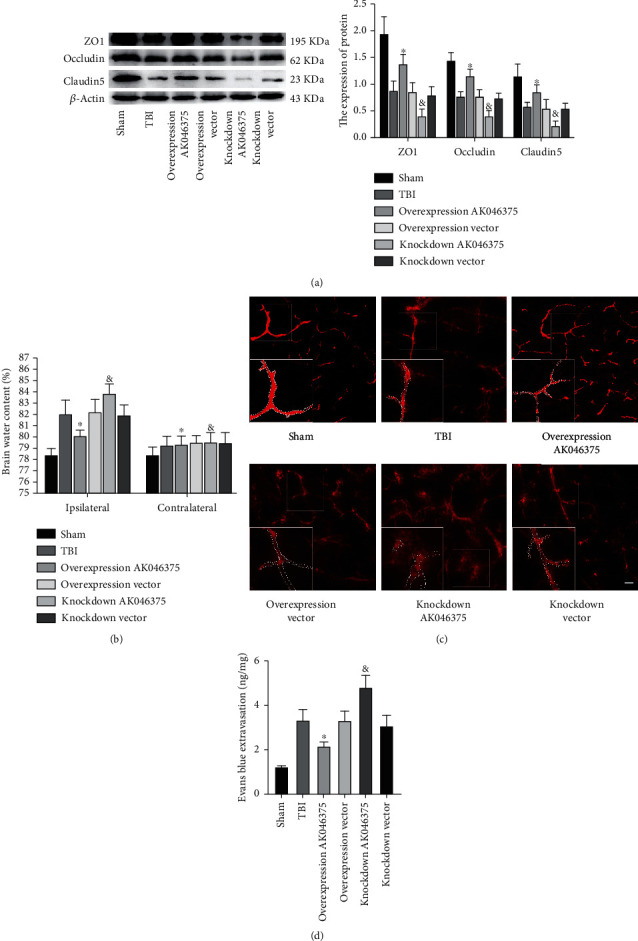
AK046375 maintains BBB integrity and decreases brain water content in mice on 7 days after TBI. (a) Western blotting results of ZO1, occludin, and claudin-5 in each group and quantification (*n* = 6/group, mean ± SD). (b) Brain water content in each group after TBI (*n* = 6/group, mean ± SD). (c, d) Fluorescence of Evans blue and Evans blue extravasation in each group around the injury site (*n* = 12/group, mean ± SD) (scale bar = 50 *μ*m, 200x) (^∗^*P* < 0.05 vs. the overexpression vector group, ^&^*P* < 0.05 vs. the knockdown vector group by one-way ANOVA).

Occludin, claudin-5, and ZO-1 are the primary protein components of the BBB, and in our previous studies, we found that TBI induced significant loss of tight junction proteins. In the current study, we found that loss of occludin, claudin-5, and ZO-1 induced by TBI were attenuated by AK046375 overexpression (*P* < 0.05, Figure 7(a)) and were aggravated by AK046375 knockdown (*P* < 0.05, Figure 7(a)).

The dry/wet weight and Evans blue methods were performed to measure BBB permeability. There was no significant difference in brain water content or the content of Evans blue in the brain among the TBI, overexpression vector, and knockdown vector groups (*P* > 0.05, Figures 7(b) and 7(d)). Brain water content and the exudation of Evans blue were reduced in the AK046375 overexpression group (*P* < 0.05, Figures 7(b) and 7(d)) but increased in the AK046375 knockdown group (*P* < 0.05, Figures 7(b) and 7(d)) compared to their respective control groups. Additionally, the fluorescence of Evans blue showed that AK046375 overexpression decreased the exudation of Evans blue from the vasculum, but AK046375 knockdown deteriorated the exudation of Evans blue (*P* < 0.05, Figure 7(c)) compared to their control groups. These results prove that AK046375 maintains the integrity of the BBB and decreases brain edema after TBI.

#### 3.4.4. AK046375 Significantly Improves Motor Function, Learning Ability, and Spatial Memory in Mice after TBI

**Figure 8:**
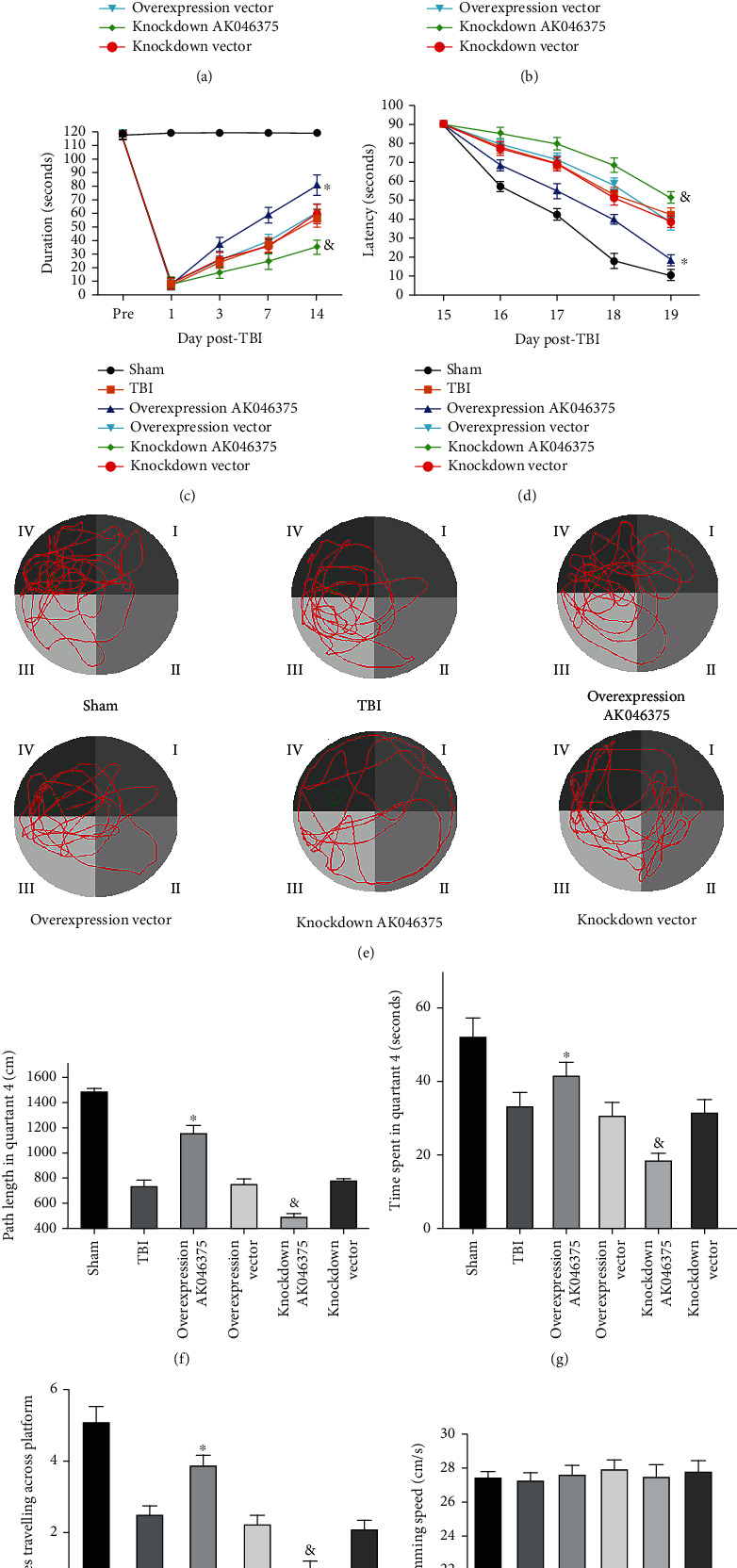
AK046375 improves motor function, learning ability, and spatial memory in mice after TBI. (a) NSS scores were detected on preoperative, 1, 3, 7, and 14 days after TBI. (b) Wire-gripping scores in each group on preoperative, 1, 3, 7, and 14 days after TBI. (c) The duration of rotarod test in each group on preoperative, 1, 3, 7, and 14 days after TBI. (d) Latency spent in quadrant 4 in cued learning performance in the Morris Water Maze (MWM) test on 15 to 19 days after TBI. (e) Swimming tracks in MWM test on 20 days after TBI. (f) Length of the swimming tracks in quadrant 4 on 20 days after TBI. (g) Time in quadrant 4 in MWM test on 20 days after TBI. (h) Times traveling across the platform in MWM test on 20 days after TBI. (i) Swimming speed in MWM test on 20 days after TBI (*n* = 8/group, mean ± SD) (^∗^*P* < 0.05 vs. the overexpression vector group, ^&^*P* < 0.05 vs. the knockdown vector group by two-way ANOVA test for NSS scores, wire-gripping scores, rotarod test, and latency spent in quadrant 4. One-way ANOVA for length of the swimming tracks in quadrant 4, time in quadrant 4, and the number of times the mice crossed over the original location of the platform on 20 days after TBI).

The NSS and water maze tests were used to evaluate whether AK046375 influenced the recovery of motor function, learning abilities, and spatial memory in mice after TBI, respectively. One, 3, 7, and 14 days after TBI, there was no significant difference in NSS scores, wire-gripping scores, and rotarod test among the TBI, overexpression vector, and knockdown vector groups (*P* > 0.05, Figures 8(a)–8(c)). NSS scores were significantly higher in the AK046375 knockdown group and lower in the AK046375 overexpression group (*P* < 0.05, Figure 8(a)) compared to their respective control groups on 3, 7, and 14 days after TBI. The wire-gripping scores and the duration of rotarod were significantly higher in AK046375 overexpression group (*P* < 0.05, Figures 8(b) and 8(c)) but lower in the AK046375 knockdown group (*P* < 0.05, Figures 8(b) and 8(c)) compared to their respective control groups. In the Morris water maze test, there was no significant difference in the latency to navigate the hidden platform on 15, 16, 17, 18, and 19 days after TBI between the TBI, overexpression vector, and knockdown vector groups (*P* > 0.05, Figure 8(d)). Besides, there was also no significant difference in swimming distance spent in quadrant 4, the time spent in quadrant 4, and the times passed over the platform location on 20 days after TBI between the TBI, overexpression vector, and knockdown vector groups (*P* > 0.05, Figures 8(f)–8(h)). Sixteen, 17, 18, and 19 days after TBI, latency was significantly decreased in the AK046375 overexpression group (*P* < 0.05, Figure 8(d)) and increased in the knockdown group (*P* < 0.05, Figure 8(d)) compared to their respective control groups. The hidden platform was removed on 20 days after TBI, the time and swimming distance spent in quadrant 4, and the times passed over the platform location were also significantly increased in the AK046375 overexpression group (*P* < 0.05, Figures 8(f)–8(h)) and decreased in the knockdown group (*P* < 0.05, Figures 8(f)–8(h)) compared to their respective control groups; there was no significant difference in the swimming speed between the TBI, overexpression vector, and knockdown vector groups (Figure 8(i)). These results demonstrated that AK046375 promotes motor function, learning ability, and spatial memory recovery in mice after TBI.

## 4. Discussion

lncRNAs are reported to regulate gene expression and chromatin structure via the decoy, scaffold and posttranscriptional effects [24]. Our pervious study demonstrated that AK046375, which is abundantly expressed in the brain, was significantly upregulated in the cerebral cortex around the TBI focus (~4-fold in contrast to preinjury levels) [8]. Our present study is aimed at interrogating the implications of increased AK046375 expression in the injured brain after TBI.

First, we applied the *in situ* hybridization to confirm the existence and subcellular location of lncRNA AK046375 in neurons and astrocytes, the most common cell types in the mammalian brain, and found that AK046375 was expressed in both the nucleus and cytoplasm but was concentrated primarily in the cytoplasm (Supplementary fig. 4). Owing to the development of RNA-seq, we observed that the MT2 gene was the most significantly altered among all differentially expressed genes in response to AK046375 overexpression. To further confirm that MT2 was significantly increased in response to AK046375 overexpression, primary neurons and astrocytes were transfected with overexpression or knockdown viruses, and we found that both MT2 mRNA and protein levels were significantly increased or decreased, respectively, illustrating that MT2 is a downstream factor in the AK046375 pathway. MT2 is a special protein with low molecular weight and high cysteine content that binds a large number of heavy metals ions [25, 26], giving it a stronger ability to scavenge free radicals than either SOD or GSH. [27–29] MT2 is highly expressed in the CNS [20] and plays a neuroprotective role against brain injury through its antioxidative effect [30, 31]. In the current study, we demonstrate that the upregulated expression of lncRNA AK046375 can significantly inhibit the overoxidative response in vitro and in vivo.

Oxidative stress, neuroinflammation, and apoptosis are common features that occur after TBI, which, in turn, compromise normal cellular functions [32]. Overloading of intracellular oxidative stress leads to mitochondrial dysfunction and activation of the mitochondria-related apoptosis pathway [33]. Once cytochrome C is released from mitochondria, caspase-3 is activated and irreversibly leads to apoptosis [34], but BCL2 inhibits activation of caspase-3 by inhibiting the release of cytochrome C from the mitochondria to the cytoplasm [35]. In our present study, the decrease of antiapoptotic proteins (BCL2) and the increase of proapoptotic proteins (Bax, cleaved-caspase-3) induced by either TBI *in vivo* or H_2_O_2_*in vitro* were partially but still significantly reversed by AK046375 overexpression treatment, which was accompanied by less TUNEL-positive staining cells, suggesting an antiapoptic effect of lncRNA AK046375. Moreover, overloading oxidative stress is one of the causes of BBB disruption [36], which, in turn, deteriorates neurological function in mice after TBI. Previous research indicated that maintaining BBB integrity after TBI improved prognosis [2, 37]. In this study, we confirmed that AK046375 overexpression attenuated the loss of tight junction proteins, brain water content, and exudation of Evans blue, all of which could be attributed to reduced oxidation levels from AK046375 treatment. Most importantly, deficits in motor function, learning ability, and spatial memory in mice after TBI were significantly attenuated by AK046375 overexpression, suggesting that AK046375 may represent a potential target that deserves more attention in TBI therapeutic research.

Here, we also go further to elucidate the possible modulating mechanism of AK046375 on MT2. It is well known that lncRNA exerts its biological functions by serving as a “sponge” for miRNA, which negatively regulate targeted mRNA expression [28]. Interestingly, bioinformatics predictions indicated that AK046375 might upregulate MT2 translation through sequestering miR-491-5p or/and miR-505-3p. Our dual-luciferase reporter assay demonstrated that only miR-491-5p directly binds to the 3′-UTR MT2 mRNA and inhibits its translation. Previous studies on miR-491-5p have primarily focused on its roles in tumor pathology, such as inhibiting tumor proliferation, motility, and metastasis by posttranscriptionally modulating target genes [31, 38, 39]. There is no evidence regarding the interaction between miR-491-5p and MT2 that could be found, and here, for the first time, we showed that miR-491-5p negatively regulates the expression of MT2 and, in turn, exacerbates oxidative stress and cellular injury induced by H_2_O_2_ treatment. Of note, due to the primary neurons being unable to endure the lipo2000 cytotoxicity, we only transfected miR-491-5p mimics and inhibitor into the primary cortical astrocytes.

To further interrogate the potential relationship between miR-491-5p and AK046375, we measured miR-491-5p expression in response to different AK046375 levels. Results revealed that miR-491-5p expression was significantly up- and downregulated when AK046375 levels were decreased and increased, respectively, suggesting that there exists a mechanism that may mediate the regulation of AK046375 on miR-491-5p. A recent theory on competing endogenous RNAs (ceRNAs) proposes that lncRNAs modulate expression of other genes by forming microRNA response elements (MREs) [40]. Given the capability of AK046375 to capture miR-491-5p and the direct binding of miR-491-5p with the 3′-UTR MT2 mRNA, we propose that AK046375 may function as an endogenous “sponge” to attract endogenous miR-491-5p, which in turn relieves the inhibition of miR-491-5p on MT2-mRNA translation and promotes more MT2 protein production. Therefore, elevated levels of AK046375 in the injured brain tissue after TBI may effectively enhance MT2 expression through inhibiting the blocking of miR-491-5p on MT2-mRNA and ameliorating oxidative stress-related injury, which would certainly inhibit apoptosis and BBB damage, promoting neuronal survival and facilitating neurological function recovery. Of note, in our current study, we only explored the binding possibility of AK046375 interacting with miR-491-5p and miR-505-3p. However, we believe, in addition to miR-491-5p, there are probably other miRNAs that are be directly captured by AK046375, and the RIP-seq method could be helpful to discern all other AK046375-binding miRNAs. Moreover, in addition to MT2, other genes may also be involved in the neuroprotective functions of AK046375. For example, mRNA levels of mt-Nd1, 3, 4, and mt-Adp8 genes were also found to be significantly altered in response to AK046375 overexpression, which also suggests a potential relationship between AK046375 and those genes. mt-Nd1, 3, 4, and mt-Adp8 are mitochondrial ATP-producing pathway-related genes, suggesting that AK046375 might also be involved in ATP production in mitochondria.

Collectively, our present study demonstrated that the increased endogenous lncRNA-AK046375 plays antioxidation roles in the injured cerebral cortex after TBI, suggesting it may represent a therapeutic target for TBI treatment. The mechanism underlying the antioxidant effect of AK046375 is likely based on enhancing MT2 expression through sequestering miR-491-5p.

## Data Availability

The data used to support the findings of this study are available from the corresponding author upon request.

## Supplementary Materials

Supplementary Materials We had acquired the full length of AK046375, and AK046375 is primarily localized in the cytoplasm; we also obtained the concentrations of H_2_O_2_ used to establish the oxidative model in primary cortical neurons and astrocytes. MT2 was the most significantly altered mRNA following AK046375 overexpression which was proved *in vivo* and *in vitro*. AK046375 could improve the primary cortical neurons and astrocytes survival under the H_2_O_2_ treatment.
