# Supplementary material for: The lncRNA-AK046375 Upregulates Metallothionein-2 by Sequestering miR-491-5p to Relieve the Brain Oxidative Stress Burden after Traumatic Brain Injury
Source: Oxid Med Cell Longev. 2022 Feb 16;2022:8188404. doi: 10.1155/2022/8188404 (PMC8865981; doi:10.1155/2022/8188404)
Supplement: Supplementary Materials — We had acquired the full length of AK046375, and AK046375 is primarily localized in the cytoplasm; we also obtained the concentrations of H2O2 used to establish the oxidative model in primary cortical neurons and astrocytes. MT2 was the most significantly altered mRNA following AK046375 overexpression which was proved in vivo and in vitro. AK046375 could improve the primary cortical neurons and astrocytes survival under the H2O2 treatment. [file 8188404.f1.docx]

**Supplementary Information**

**Materials and methods**

**Rapid amplification of cDNA ends (RACE) analysis**

The RACE analysis was used to confirm that the lncRNA AK046375 is truly one transcript and obtain the full length of the lncRNA AK046375. In briefly, Trizol reagent (Takara, Japan) was used to extract the RNA form the brain tissue around the injury site at 24H after TBI. The RNA primers for 5′-RACE (forward: GTGTGCTGGCCATATCCCTTGAGCCAGAA. reverse: CGACCC AATACTCTCCGCTATAAAGGTC), 3′-RACE (forward: CTGGGGCAATGCCTTCTTGACTCA TTCC. reverse: GCTGCCCTGGCTATGAACTTGAACCG) and the intermediate sequence (forward: GAAGATCGACGAGAGATCGGTTT. reverse: CTCTGGGGTCAGTCTGGAAA) of ak046375 were constructed, the 5′- ,3′ - and the intermediate sequence of lncRNA AK046375 were respectively amplificated to run the 1.5% agarose gel electrophoresis by the GeneRacer^TM^ Kit (Invitrogen, America), the target gels were obtained and connected to the pGM-T vectors (Sangon Biotech, Shanghai, China), the vectors were transfected to DH5α cell (Sangon Biotech, Shanghai, China) respectively and sequencing analysis was performed. Integrating the above results, we obtained the full-length sequence of lncRNA AK046375.

**LncRNA AK046375 overexpression in HT22 cells and mRNA sequencing** The detailed protocols which were used to infected HT22 cells with virus has been previously described. In brief, HT22 cells were infected with either AK046375 overexpression adenoviruses or overexpression vector, and then the total mRNAs were harvested on the 5 days after the infection. The efficiency of AK046375 overexpressing was measured using RT-PCR. Two groups were established, 1) an AK046375 overexpression group that was infected with AK046375 overexpression adenoviruses, 2) an overexpression vector group that was infected with overexpression vector adenoviruses. The total RNA collected from cells were used to perform RNA-seq on a HiSeq 2500 system by next-generation sequencing techniques. The sequencing data were filtered and analyzed based on the detailed procedures reported in our previous study. Significant changes in the transcript levels were identified using the following criteria: |log _2_^(fold change)^ | > 1, *P* < 0.05.

**Fluorescent in situ hybridization analysis**

To detected the subcellular localization of lncRNA-AK046375 in primary cortical neurons and astrocytes, cells were fixed in 4% paraformaldehyde, digested with proteinase K, hybridized with the probes of lncRNA AK046375 (5’-FAM-GTGAGAATAATGAACACTCCAACCAGCGTTTGTA AGG) at 42 °C for 12h, incubated with the primary mouse anti-β3-tubulin antibody (1:50) (Santa Cruz, America) or mouse anti-glial fibrillary acidic protein (GFAP) antibody (1:400) (R&D, America) at 4°C for overnight, then incubated with the secondary antibody (Dylight 594, goat anti-mouse IgG) (Abbkine, America) at 37 °C for 60 min, and finally incubated with DAPI for 5 mins at room temperature. The images were taken by microscopy (Carl Zeiss AG, Germany).

**DCFH-DA assay**

The reactive oxygen species assay kit (Beyotime biotechnology, Shanghai, China) were used to assess the expression of reactive oxygen species (ROS) in cells under different treated conditions following the procedures in the product instruction. After the preliminary experiment was ready, 100 µL of serum free medium containing DCFH-DA working buffer (1:1000) were added into each well and incubated at 37 ℃ for 20 min, and then samples were imaged on the confocal microscopy (excitation wavelength: 488nm, emission wavelength: 525 nm), and the fluorescence intensity was quantified by Image pro plus.

**Oxidative stress model in vitro**

For primary cortical neuron oxidative stress model, the concentration of H2O2 which was used to induce the neuronal oxidative stress model was referred to a published study and modified accordingly. In briefly, the cells were exposed to the gradient concentrations of exogenous H2O2 (0, 50, 100, 200, 400, and 800 μmol/l) for 12 h, and then followed by CCK8 assay and DCFH-DA assay. For primary astrocytes oxidative stress model, the different gradient concentrations of exogenous H2O2 (0, 50, 100, 200, 400, and 800 μmol/l) for 3h were used to induce astrocytes oxidative stress model, and followed by CCK8 assay or DCFH-DA assay.

**Extracted cytosolic fraction and mitochondrial fraction**

The cytosolic fraction and mitochondrial fraction of the H_2_0_2_ treated cells (primary cortical neurons and astrocytes) or brain tissues after TBI were extracted with a mitochondria isolation kit (Beyotime biotechnology, Shanghai, China), 500 µl mitochondria isolation reagent was added into each sample, followed by homogenization and incubation on ice for 15 min. The samples were then vortexed 15 seconds, centrifugated for 10 min (600g/min) at 4 ℃, and then transferred the supernatants to another microcentrifuge tubes, centrifuged for 10 min (11000g/min) at 4 ℃, the precipitation was mitochondria fraction and the supernatants were cytosolic fraction.

**Figure legends**

**Supplementary figure 1. The full length of AK046375 in brain tissue around the injury site at 24H after TBI.** 5′ -RACE and 3′ -RACE of lncRNA AK046375, the intermediate sequence amplification of lncRNA AK046375, and the full-length sequence of lncRNA AK046375.


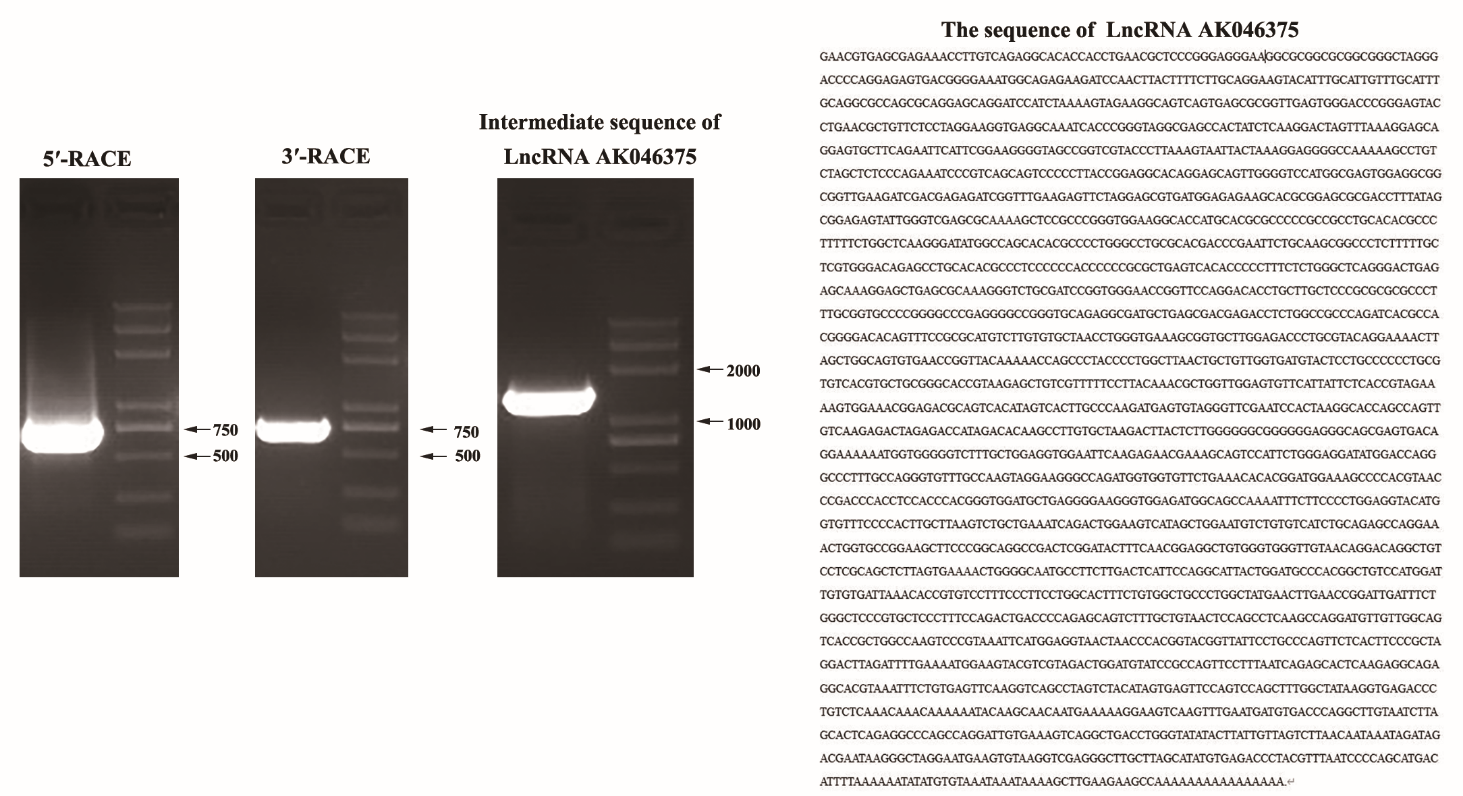


**Supplementary figure 2. AK046375 significantly upregulates the transcription of MT2 in HT22 cells.** (A-a) Quantitative PCR analysis of AK046375 levels after transfection with AK046375 and overexpression vector adenovirus in HT22 cells (n = 4/group, mean ± SD). (A-b) RNA-sequencing indicates that 1342 mRNA are significantly changed in accordance with AK046375 overexpression adenovirus transfection (717 upregulated and 615 downregulated) by the screening criteria (|log_2_^(fold change)^|>1, *P<*0.05), all raw data were submitted to the GEO database (ID number: GSE103353). (B) Western blotting results of MT2 in each group (left) and quantification (right) (n = 6/group, mean ± SD) (^*^*P*<0.05 vs the overexpression vector group by t-test).


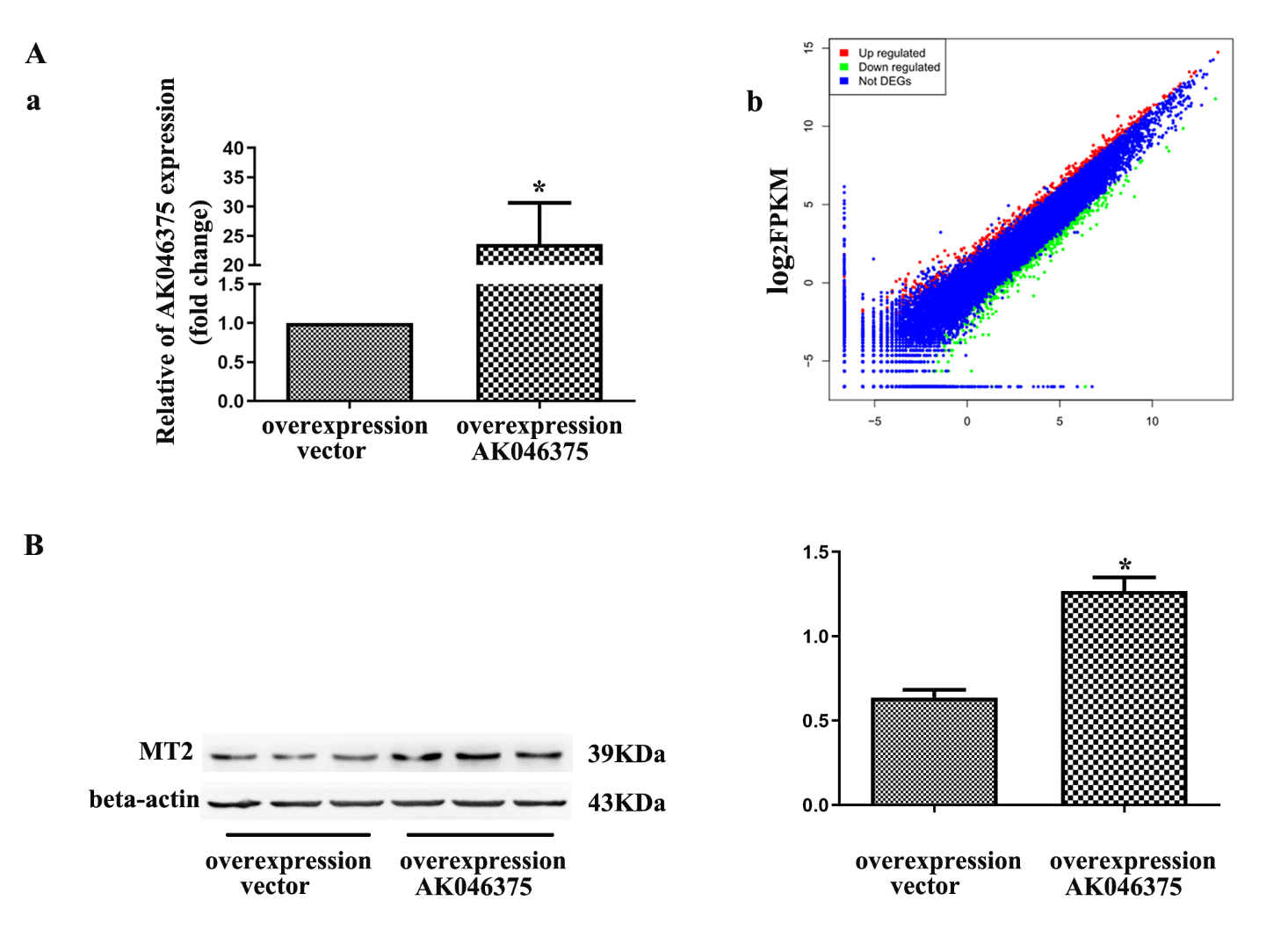


**Supplementary figure 3. Concentrations of H2O2 used to establish the oxidative model in primary cortical neurons and astrocytes.** 1) Neuron (A) and astrocyte (C) viability in each group. 2) Fluorescence intensity of ROS in neurons (scale bar=50µm) (B) and astrocytes (scale bar=100µm) (D) induced by H2O2 (200 µm × 12 h for neurons, 400 µm × 3 h for astrocytes) (left) and quantification (right) (n=6/group, mean ± SD, **P*<0.05 vs the non-treated group by t-test).


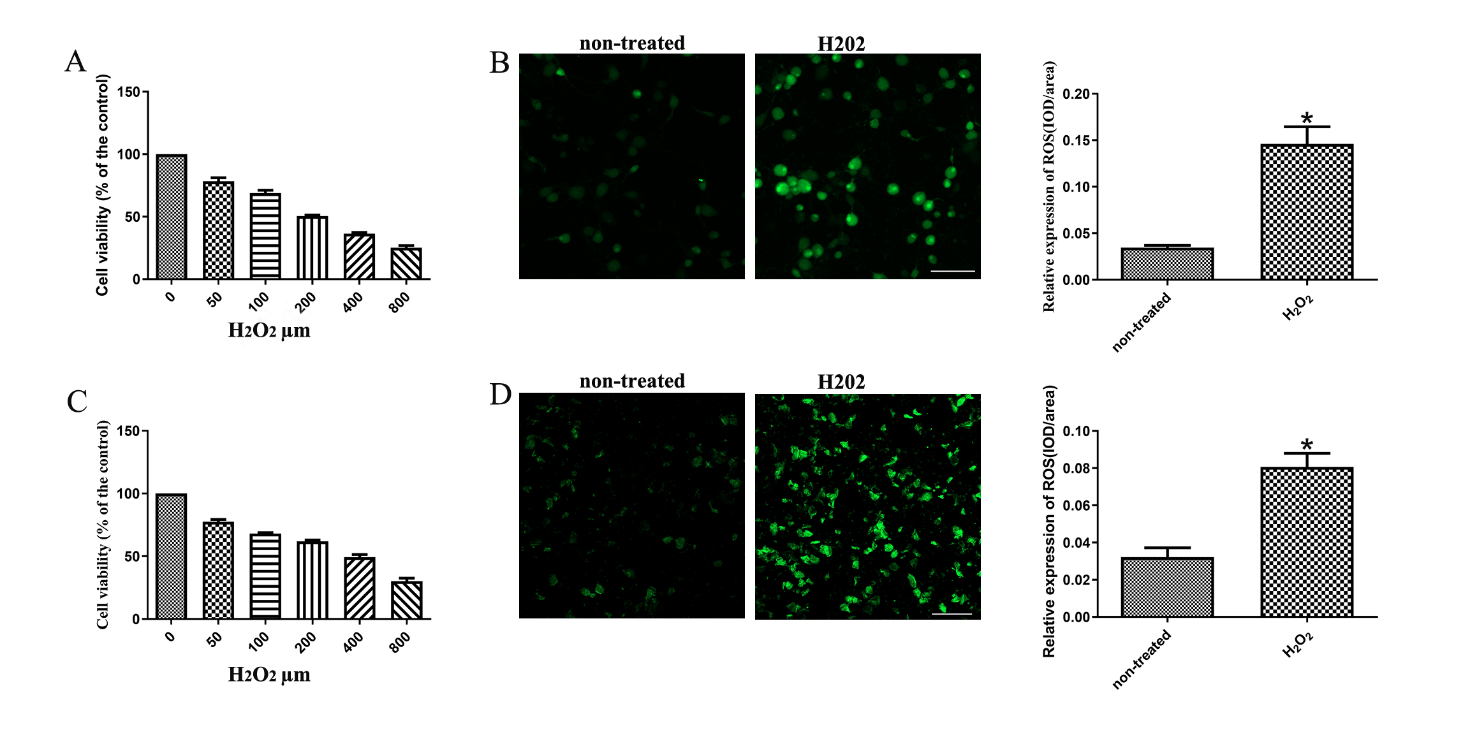


**Supplementary figure 4. AK046375 is primarily localized in the cytoplasm.** The subcellular localization of AK046375 in primary cortical neurons (A) and astrocytes (B) visualized by AK046375 fluorescence *in situ* hybridization (FISH) probes. β3-tubulin is a neuronal marker, and GFAP is an astrocytic marker (scale bar=20 µm, 800× for neurons, scale bar=50 µm, 400× for astrocytes).


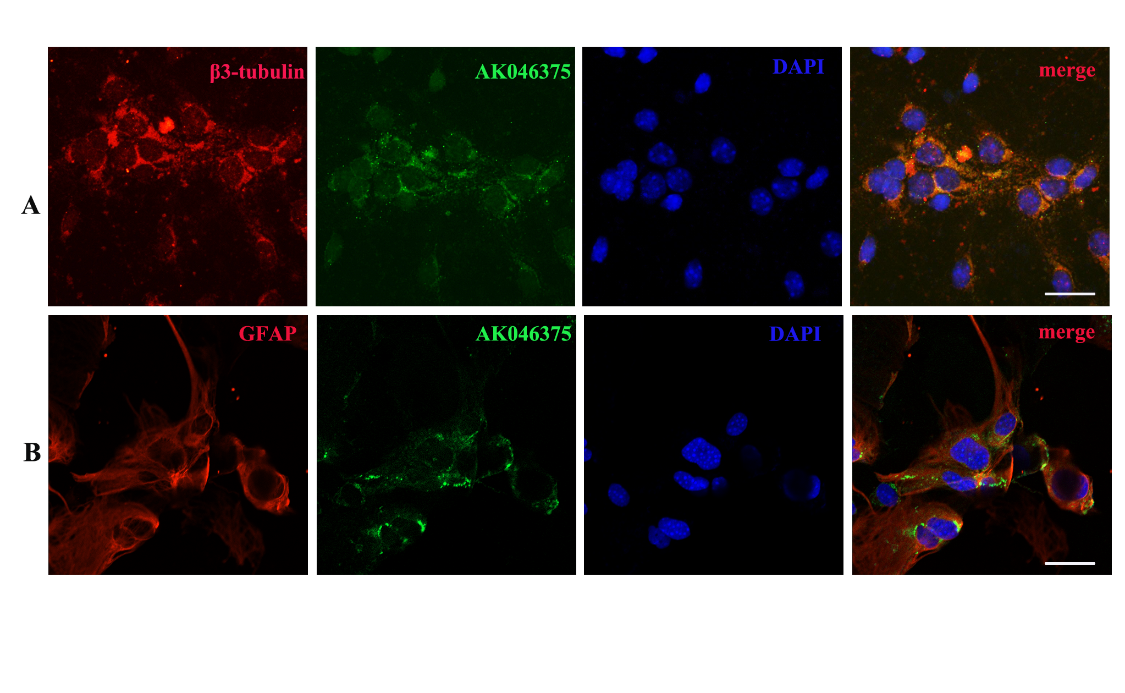


**Supplementary figure 5. AK046375 increases survival in primary cortical neurons and astrocytes.** (A) Cell viability of primary neurons (left) and LDH in neuronal media (right) in each group. (B) Cell viability of primary astrocytes (left) and LDH in astrocytic media (right) in each group (n=6/group, mean ± SD, **P*<0.05 vs the overexpression vector+H2O2 group, ^&^*P*<0.05 vs the knockdown-vector+H2O2 group by one-way ANOVA).


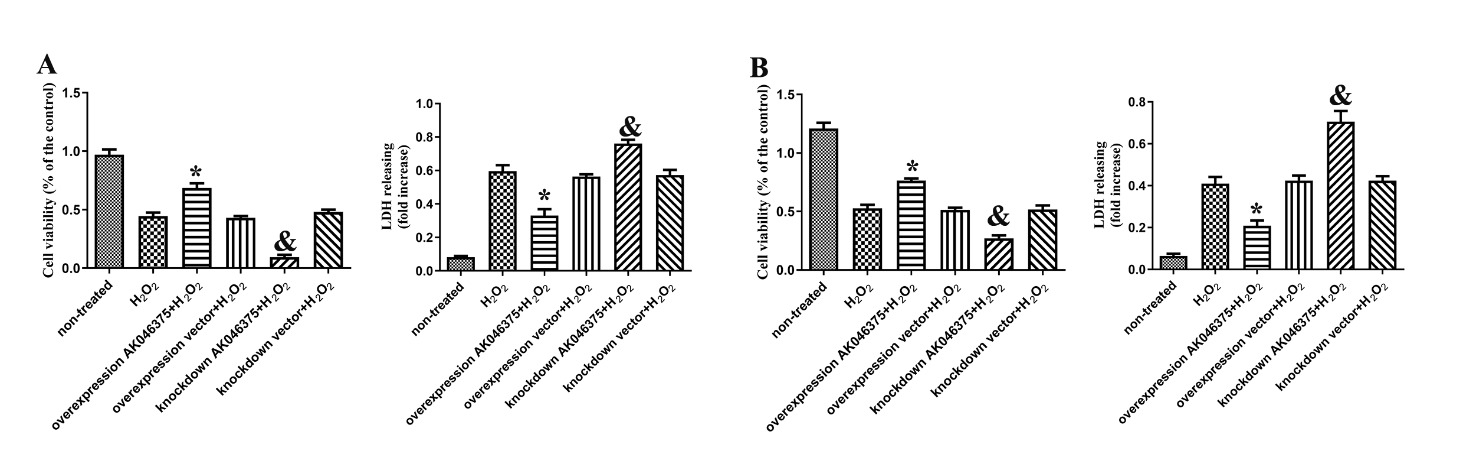


**Supplementary figure 6. AK046375 upregulates expression of MT2 *in vivo*.** (A) Quantitative PCR analysis of AK046375 levels in mouse cortex in each group. (B, C) Quantitative PCR analysis of mRNA levels and Western blotting results of MT2 in each group (n=4/group, mean ± SD, ^*^*P*<0.05 vs overexpression vector group, ^&^*P*<0.05 vs the knockdown vector group by one-way ANOVA).


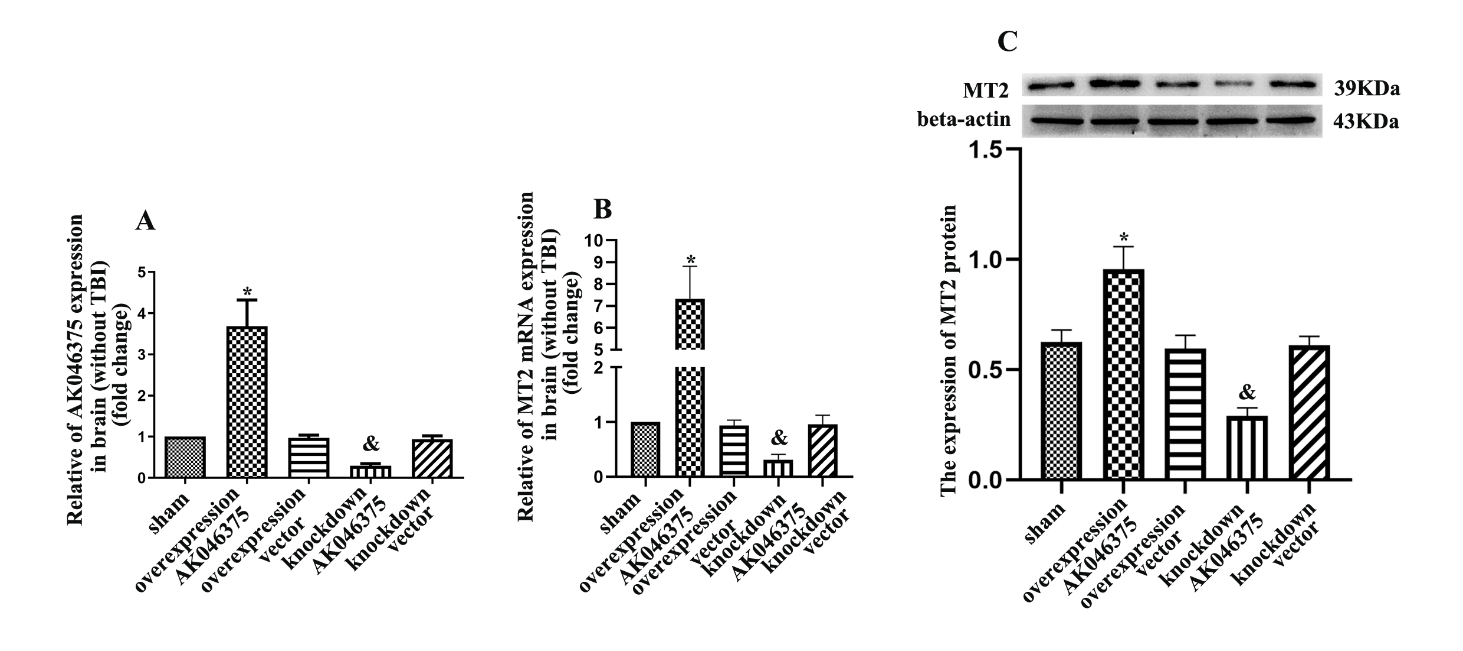


**Supplementary Table 1. The most significantly altered 10 mRNAs in response to AK046375 overexpression in HT22 cells.** MT2 was the most significantly altered mRNA following AK046375 overexpression.

| **Gene name** |  | **Ensemble ID** | **Normal reads** | **AK046375 over-expression** | **Type** | **fold change** | **log_2_^（fold change）^** | **P-value** |
| --- | --- | --- | --- | --- | --- | --- | --- | --- |
| **MT2** |  | **ENSMUSG00000031762** | **624.06** | **3675.71** | **up** | **5.89** | **1.88** | **0.00** |
| **mt-Nd3** |  | **ENSMUSG00000064360** | **282.83** | **1615.05** | **up** | **5.71** | **2.72** | **0.00** |
| **mt-Atp8** |  | **ENSMUSG00000064356** | **1909.95** | **341.79** | **down** | **5.6** | **2.42** | **0.00** |
| **mt-Nd1** |  | **ENSMUSG00000064341** | **162.1** | **681.16** | **up** | **4.2** | **2.33** | **0.00** |
| **mt-Nd4** |  | **ENSMUSG00000064363** | **112.96** | **442.84** | **up** | **3.92** | **2.23** | **0.00** |
| **Nhp2** |  | **ENSMUSG00000001056** | **165.95** | **628.46** | **up** | **3.79** | **2.17** | **0.00** |
| **Uqcrq** |  | **ENSMUSG00000044894** | **450.76** | **1543.2** | **up** | **3.43** | **2.02** | **0.00** |
| **Mrpl20** |  | **ENSMUSG00000029066** | **318.9** | **1051.91** | **up** | **3.29** | **1.96** | **0.00** |
| **Snrpf** |  | **ENSMUSG00000020018** | **176.82** | **552.77** | **up** | **3.13** | **1.89** | **0.00** |
| **Polr2f** |  | **ENSMUSG00000033020** | **466.55** | **1462.14** | **up** | **3.14** | **1.86** | **0.00** |
